# Sequence-based evaluation of promoter context for prediction of transcription start sites in Arabidopsis and rice

**DOI:** 10.1038/s41598-022-11169-w

**Published:** 2022-04-28

**Authors:** Tosei Hiratsuka, Yuko Makita, Yoshiharu Y. Yamamoto

**Affiliations:** 1 Graduate School of Natural Science and Technology, Gifu University, Yanagido 1-1, Gifu, 501-1193 Japan; 2 RIKEN Center for Sustainable Resource Science, Hirosue-Cho 1-7-22, Tsurumi-ku, Yokohama, Kanagawa 230-0045 Japan; 3 Faculty of Applied Biological Sciences, Gifu Uniersity, Yanagido 1-1, Gifu, 501-1193 Japan; 4 Present Address: Faculty of Engineering, Maebashi Institute of Technology, Kamisadori 460-1, Maebashi, Gunma 371-0816 Japan

**Keywords:** Plant molecular biology, Plant genetics

## Abstract

Genes are transcribed from transcription start sites (TSSs), and their position in a genome is strictly controlled to avoid mis-expression of undesired regions. In this study, we designed and developed a methodology for the evaluation of promoter context, which detects proximal promoter regions from − 200 to − 60 bp relative to a TSS, in Arabidopsis and rice genomes. The method positively evaluates spacer sequences and Regulatory Element Groups, but not core promoter elements like TATA boxes, and is able to predict the position of a TSS within a width of 200 bp. An important feature of the evaluation/prediction method is its independence of the core promoter elements, which was demonstrated by successful prediction of all the TATA, GA, and coreless types of promoters without notable differences in the accuracy of prediction. The positive relationship identified between the evaluation scores and gene expression levels suggests that this method is useful for the evaluation of promoter maturity.

## Introduction

Promoters determine not only the frequency and timing of transcription, but the position and direction of transcription start sites (TSSs). Decoding this information from the nucleotide sequence is one of the most important goals of functional genomics.

TSS of a gene used to be determined as the most upstream end of several full-length cDNA clones covering the downstream gene body, which was reflected to gene models. Development of TSS-seq supported by the emerged NGS technology enabled TSS mapping with higher scales^1,2^, revealing that TSS of a gene is made of a cluster with a width of tens of bp and that multiple clusters are associated with a gene. Accordingly, the most major TSS, or the peak TSS, of the top cluster is considered as the representative transcription start site of a gene^3^. This data is indispensable for extraction of functional elements and likewise recognition of promoter structure of each gene^4^.

During the analysis, the distribution of TSS was found to be of hierarchical orders^5^, therefore, they can be recognized using these orders. In mammalian genomes, the orders range from a gene-wide length of about 10 kbp; to a range of clusters of TSSs spanning ~ 100 bp; and the finest range which determines distribution of TSSs in a cluster, of one bp^5^. In addition, to these three orders, the distribution of TSS is regulated by chromatin structure, often accompanied by epigenetic modulation in chromosomal to local ranges^6^. The emergence of a TSS is thus a result of multi-level regulation.

The finest order of regulation, which we call local-range regulation, is known to be determined by a well-known consensus sequence motif called Initiator (Inr)^7^. This motif is found in a limited number of genic promoters, and is therefore not applicable to the majority of promoters. Genome-wide TSS analysis has revealed some rules which appear to be followed in mammals^5,8^ and higher plants^9^. The latter, called the YR Rule, is a relaxed form of the Inr motif, and covers the majority of *Arabidopsis* promoters. The major factor influencing the position and direction of TSSs had been thought to be the presence of the TATA box, a core promoter element found in yeast, *Drosophila,* higher plants, mammals, and many other eukaryotes^7^. However, large-scale promoter analyses have revealed that 32% of promoters in humans^1^ and 25% in *Arabidopsis*^9^ contain TATA boxes, demonstrating that TATA-positive promoters are in the minority in both animals and plants.

The finding of TATA-less promoters led to the identification of other core elements among these promoters, including DPE in *Drosophila*^7^, and GA and CA elements in higher plants^4,9^. Mammalian TATA-less promoters have been found to be associated with CpG islands^1^. This group of sequences is related to several elements, including Sp1^4,8^, but because of its sequence diversity, the CpG islands themselves are not thought to be *cis*-element for a specific DNA-binding protein. Therefore, they could be a core promoter element modifying the DNA structure to make it suitable for transcriptional initiation.

The core elements are believed to be factors in the middle-range (~ 100 bp) TSS determination. It is not known whether the core elements alone are enough to restrict TSSs within the middle range, or whether additional determinants are required. Middle-range determinants in coreless promoters, which account for 28% of *Arabidopsis* promoters^9^, are poorly understood. Therefore, it is not clear whether there is a general determinant for the middle-range restriction of TSSs which is applicable to both coreless and core-containing promoters.

We are interested in the evaluation of promoter context with a span of ~ 1 kb region. We hypothesize that in addition to the core promoter elements it is also related to the middle-range TSS distribution. This topic is not well understood, but we assume it is a middle-range constituent of promoters if it does exist. If it does, the detection and assessment of the promoter context can assist in the prediction of promoter positions for coreless type promoters. Their methodology has the potential to measure promoter maturity, which distinguishes newly emerged promoters from long-existing ones without taking sequence conservation into consideration. Our work on the evaluation of sequences in the proximal and distal promoter regions resulted in the establishment of a successful measure of the promoter context in a core-type independent manner.

TSS-seq is an established, but laborious and cost-consuming methodology to cover the majority of genes in a genome. Although TSS info is indispensable for recognition of promoter structure, which is highly related to its expression profile, its data in higher plants is currently available only for Arabidopsis^3,9,10^ and maize^11^. Therefore, it is not practical to expect it from minor plant and crop species and also ecotypes and cultivars even in model plant species, such as Arabidopsis and rice. This situation can be relieved by development of sequence-based TSS prediction methods which does not require any experimental costs.

## Results

### Promoter context in Arabidopsis

**Figure 1:**
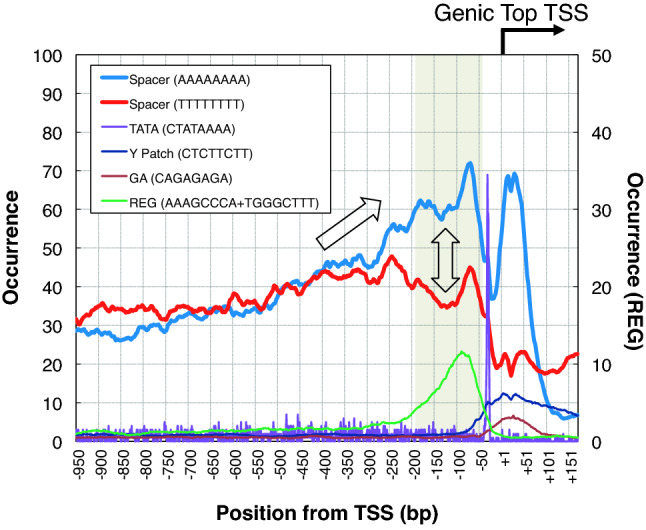
Distribution profiles of several promoter constituents. Occurrence of the octamers (Spacer, TATA, Y Patch, GA, and REG) according to the promoter position. Spacers are defined as octamers highly present in the promoter region and not core elements (TATA, GA, CA, or Y Patch) or REG. REG means Regulatory Element Group, and is identified according to its localization profile (LDSS) in the promoter region. Data of promoters for 21,673 protein-coding genes of *Arabidopsis* was summed and subjected to smoothing with a bin of 51 bp width except for the TATA octamer.

Clusters of TSSs, which correspond with promoters, are found in various positions in relation to the gene structure in the *Arabidopsis* genome. These include Genic Top, Genic Companion, Intragenic, Antisense, and Orphan^3,9^. In this work, we focused on Genic Top promoters which are the primary determinant of expression for protein-coding genes^3^. In our previous studies, analysis of the localization profile of each octamer sequence along the promoter region of the Genic Top type revealed several distinct groups of promoter constituents, including core promoter elements, Regulatory Element Groups (REGs), Inr, and some other sequences involved in translational initiation^9,12^. We identified possible spacer sequences which appear at the highest frequencies among all the octamer sequences in the promoter region, and have no known functions. Figure 1 shows the distribution profiles of octamers of several types of promoter constituents around the promoter region in the *Arabidopsis* genome. The core elements, TATA, Y Patch, and GA, are localized around the TSS, and the REG upstream of the core elements with a peak at around -100 bp, as reported previously^12^. A spacer AAAAAAAA in the figure is the most frequently observed octamer in the promoter region, and shows no match with any functional promoter elements, such as the core elements and REG. This spacer has a peak upstream of TATA, at around − 70 bp, and its occurrence gradually decreases in the upstream direction. Its frequency drops after + 51 bp, suggesting that the octamer does not occur preferentially in the coding region. Overall, the octamer preferentially localizes at the proximal promoter region. Another spacer, TTTTTTTT, shows a similar occurrence to AAAAAAAA in the distal promoter region, but has a very different distribution at the proximal region. Because these two octamers are complementary, the preferential occurrence of AAAAAAAA over TTTTTTTT produces strand bias at the proximal promoter region. These profiles may suggest that there is some promoter context elevating in the proximal promoter region over the distal region in a strand-specific manner.

In order to produce a measure of promoter context, we developed several score tables for all possible octamers, which reflects the occurrence of the octamers. One type is the Intergenic Index (IGI). IGI200_60 is a score table reflecting the occurrence of elements in a range from − 200 to − 60 bp, and the score is normalized to make the score zero for octamers with neutral occurrence. A positive score indicates more frequent occurrence than that of the neutral octamers in the region. The flow of the calculation is illustrated in Supplemental Figure S1.

We prepared another type of score, the Promoter Index (PRI), by subtracting the IGI of the distal promoter region from that of the proximal region. A high PRI is an indication of a proximal promoter region. The examination of several regions for the distal promoter within − 800 to − 200 bp revealed that a region from − 750 to − 450 bp gave the best results in TSS prediction (Supplemental Figure S2). Therefore we set PRI as "IGI200_60—IGI750_450" and it is expressed as PRI200_60-750_450, or PRI.

**Figure 2:**
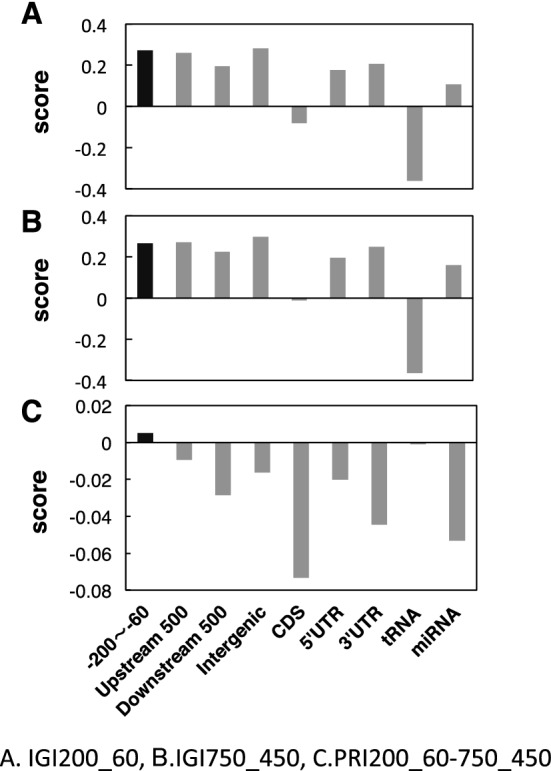
Score of genic domains and transcribed region of a few gene categories. Evaluated sequences of the categories were obtained from TAIR10. (**A**) Intergenic Index (IGI) prepared with the region from − 200 to − 60 bp. (**B**) Integenic Index (IGI) prepared with the region from − 750 to − 450 bp. (**C**) Promoter Index (PRI) prepared by the subtraction of IGI750_450 from IGI200_60. Categories giving positive scores in the three indices are highlighted in black.

Figure 2 shows the specificity of IGI and PRI in several genic regions and the transcribed region of tRNA and miRNA. Two IGI (Fig. 2A,B), proximal (IGI200_60) and distal (IGI750_450) gave similar profiles, giving positive scores for intergenic regions (− 200 to − 60, Upstream 500, Downstream 500, and Intergenic), as well as the 5' UTR, 3 'UTR, and miRNA. These results show frequently appearing sequences in the promoter region are also preferred in the UTR and intergenic regions, but not in the CDS. In contrast, PRI (Fig. 2C) had positive scores only for − 200 to − 60, and negative scores for Upstream 500, Downstream 500, and Intergenic, in addition to CDS. These results demonstrate the high specificity of PRI to the proximal promoter region.

**Figure 3:**
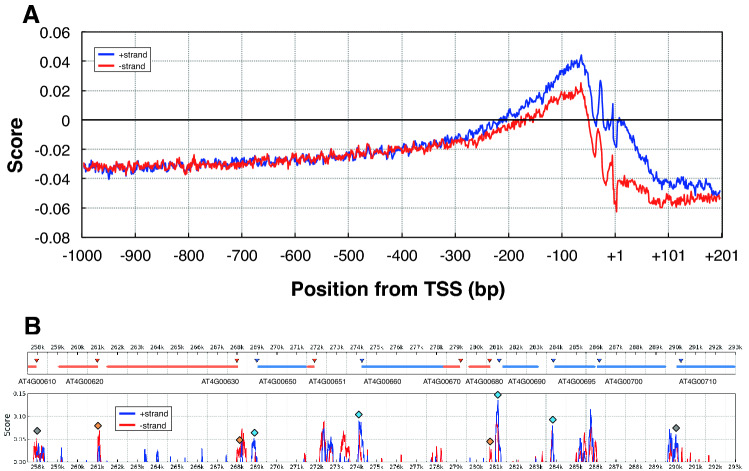
PRI along the promoter region. (**A**) Average of 21,393 Arabidopsis promoters for protein-coding genes for both strands (+ and −). (**B**) A part of Chr 1 is scanned with PRI. Upper panel shows gene models (blue = forward, red = reverse). Intron–exon information is omitted. Triangles at the head of each gene model show the position of the Genic Top TSS. Lower panel shows PRI scores. Scores were subjected to smoothing with a bin of 151 bp width. Negative scores are no shown. PRI peaks corresponding to actual TSS are shown by rhombuses (blue = forward, red = reverse).

The distribution profile of PRI around the promoter region is shown in Fig. 3A. The score of the + strand with respect to gene direction gave positive value only within the area from − 200 to + 1, with a peak around − 70, indicating the high specificity of PRI around the proximal promoter region. The other strand (-strand) also shows a similar profile, but the peak height is lower than that of the plus strand. There was a negligible difference between both strands upstream of − 300 bp, but the plus strand started to get higher from − 300 bp to + 201, indicating that the plus strand has higher PRI scores than the minus strand. These results revealed strand bias around the TSS, including the proximal promoter region, and preferential detection of the plus strand by PRI, suggesting that PRI can predict not only the proximal promoter region but the direction of the promoter.

Figure 3B shows the PRI score for both strands in an approximately 50 kb region of chromosome 4. The upper panel shows the simplified gene models, and the lower panel shows the PRI score. The experimentally identified Genic Top TSS for each gene model is shown as a triangle in the upper panel. There were two more TSS clusters for each gene on average, and likewise Intragenic, Antisense, and intergenic Orphan TSS clusters^3^, but they are not shown in the panel. The lower panel shows the PRI score in the region, and a PRI peak which corresponds to a Genic Top TSS is expressed as a rhombus. These results predicted the Genic Top TSS with very low noise, demonstrating that PRI produces accurate prediction of TSS.

### Promoter prediction using PRI

**Figure 4:**
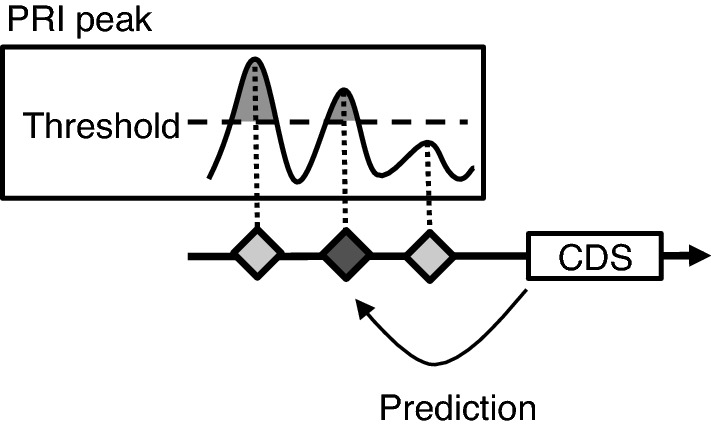
Gene model-associated TSS prediction for Genic Top promoters. One TSS is predicted for a gene. PRI peaks were searched for from the head of CDS in the upstream direction. The first peak over a threshold was selected as the predicted TSS.

Given the high specificity of PRI, we next tried prediction of promoter positions based on the genome sequence. Among the various types of promoters with respect to position and orientation of the gene, our major focus was on the Genic Top type, which has a promoter context of around 1 kb long, and is the primary driver of gene expression^3^. Our procedure for the prediction of Genic Top TSS is illustrated in Fig. 4. PRI peaks are searched for starting from the 5' end of the CDS toward the upstream, and the first peak above a threshold is selected. A region from + 1 to + 200 bp from the selected PRI peak is the predicted area for TSS. Therefore, TSS prediction in this study is not pinpoint, and occurs in an area with a width of 200 bp.

We examined effect of the threshold used on sensitivity and precision of TSS prediction in *Arabidopsis*. Evaluation of the harmonic mean (F) of the two factors examined indicated that 0.0 gave the best prediction score (Supplemental Figure S3). Therefore, we set the threshold for TSS prediction in Arabidopsis to 0.0.

We examined distance between CDS and the predicted TSS. As shown in Supplemental Figure S4A, majority of the predicted TSS appeared within 100 bp from the CDS, and as the distance lengthened, the number of promoters decreased. Some predicted promoters, however, were more than 1,000 bp from the CDS. The distance from CDSs and experimentally identified TSSs has a very similar profile to the one produced using prediction (Panel B). The comparison revealed that our prediction does not favor short distances from the CDS, but is neutral with respect to the distance.

**Figure 5:**
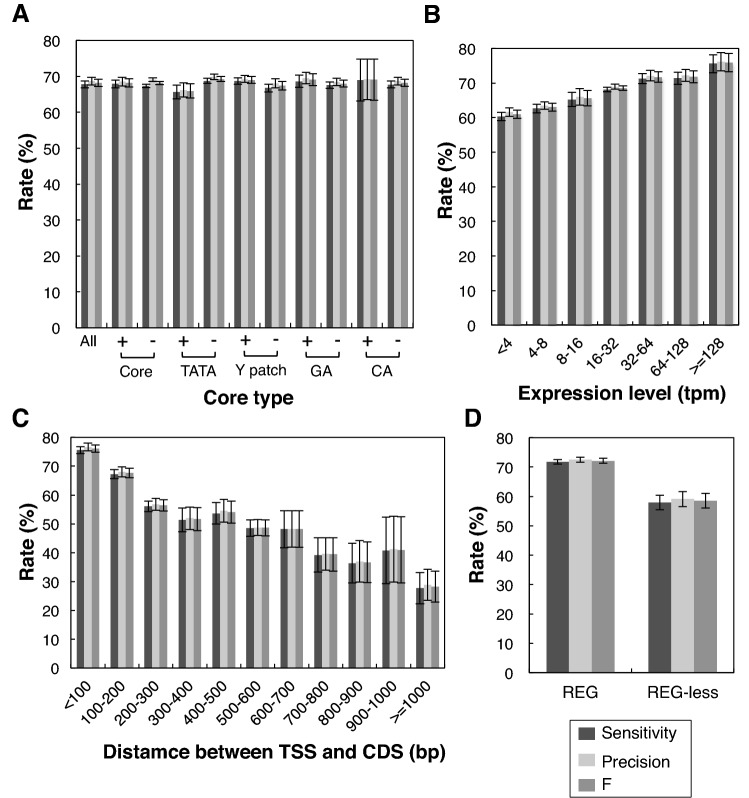
TSS prediction for various fractions of Genic Top promoters. Prediction was made for each chromosome using prepared PRI tables excluding the corresponding chromosome. The average and SD of data from five chromosomes is shown. Sensitivity, precision, and F are "true positives/ positives", "true positives/predictions", and their harmonic mean, respectively. (**A**) Prediction scores according to the core types. Promoters containing an indicated core element are compared with the rest. The definition of core elements is as described by Yamamoto et al., 2007. Core means any of core elements, TATA, Y Patch, GA Element, or CA Element. (**B**) Prediction scores according to the expression levels. Expression data of TSS-Seq were used. (**C**) Prediction scores according to the distance between TSS and CDS. The TSSs were experimentally determined Top Genic TSSs. (**D**) Prediction scores according to the presence of REG. REG (Regulatory Element Group) is not a core element but a subset of *cis*-elements (Yamamoto et al., 2007).

We then evaluated effect of several promoter characteristics on TSS prediction. Figure 5A shows the effects of the core promoter types. Promoters containing any core elements, including TATA, Y Patch, GA, and CA, and coreless promoters all showed high sensitivity, and precisions around 70%, and no significant differences were observed in the prediction scores. Therefore, the presence or absence of TA, Y Patch, GA Elements, and CA Elements did not make a significant difference to the prediction scores. These results clearly demonstrate the core type-independence of our prediction, which is one of our goals of this study.

Panel B in Fig. 5 shows the relationship between the expression level and the prediction score. The least strongly expressing promoters (< 4 tpm) had high scores of around 60% for sensitivity and precision, and they increased moderately with the elevation of expression level, showing a clear positive correlation. Assuming that promoters with high expression levels are more mature than those with low expression, this correlation suggests that PRI can be used to estimate the maturation level of promoters by evaluation of the promoter context.

The effect of the distance between a CDS and a TSS is shown in Panel C. The graph shows a moderate reduction in the prediction scores as the length between the TSS and CDS increases. Panel D shows a comparison between REG-containing and REG-less promoters. REG-containing promoters had higher prediction rates than REG-less promoters. These results are understandable, because REGs are supposed to give high scores (Fig. 1).

In summary, our prediction produced high sensitivity and precision, regardless of the core promoter types, and there were moderate differences in the score according to the expression level and the presence of REGs.

### Application to rice promoters

We applied our methodology to promoters in rice. Using the scoring parameters established for *Arabidopsis*, rice promoter sequences based on our TSS data were used for the preparation of IGI and PRI tables.

**Figure 6:**
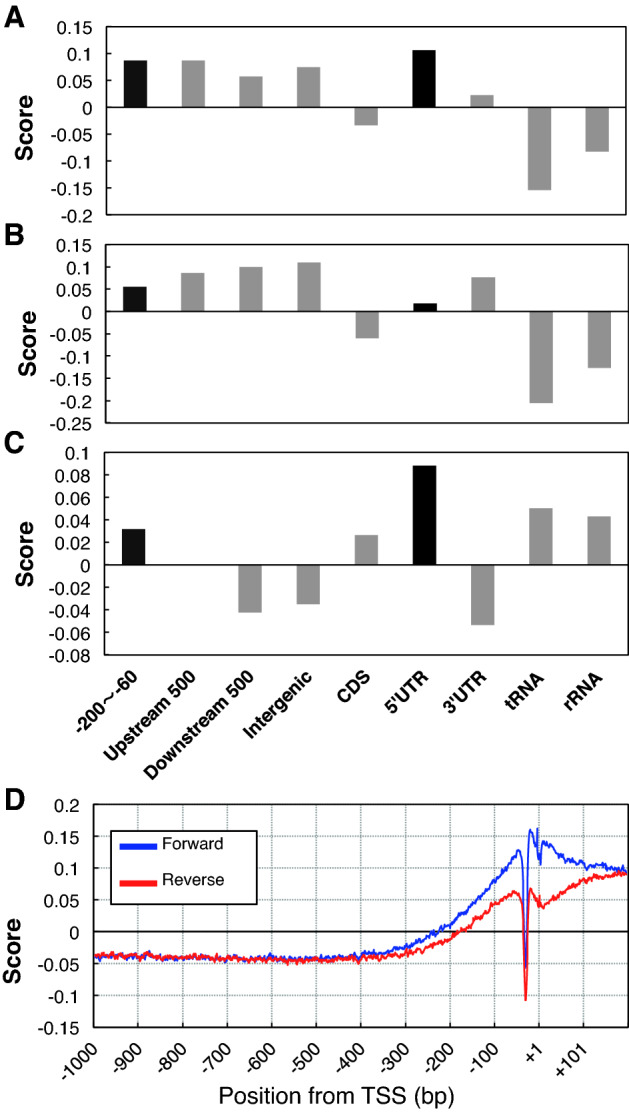
Specificity of rice PRI. (**A**) to (**C**). Scores of genic domains and transcribed region of a few gene categories are shown. The number of sequences used are: − 200 to − 60, Upstream 500, and Downstream 500 = 11.325; Intergenic = 11,666; CDS = 14,679; 5' UTR = 8,443; tRNA = 156; rRNA = 3. Sequences of − 200 to − 60, Upstream 500, Downstream 500, tRNA, and rRNA were obtained from RAPDB, and the others from MSU7. (**A**) IGI200_60, (**B**) IGI750_450, (**C**) PRI200_60-750_450. Regions giving positive scores in all three IGI/PRI are highlighted in black. (**D**) PRI along the promoter region. Average of Genic Top promoters is shown.

Figure 6 shows the average of IGI200_60 (Panel A), IGI750_450 (Panel B), and PRI200,_60-750_450 (Panel C) for various genic regions shown in the figure. Genic domains giving positive values for all the three indices are not restricted to − 200 to − 60, but the 5' UTR is also included, revealing a lower specificity of the PRI in rice than in *Arabidopsis* (Fig. 2).

Panel D shows the trends in PRI around the promoter regions. The scores of the region upstream of -250 were negative, and positive scores were found in the proximal promoter region (− 250 to + 1) and the downstream region (+ 1 to + 200), a finding which is consistent with the results shown in Panel C. The peak position of the rice PRI was around − 20, which shifted toward the downstream side compared with the peak position in Arabidopsis (− 60, Fig. 3A). In rice, the score moderately decreased toward the downstream side, which is also different from the rather steep decrease observed in *Arabidopsis* (Fig. 3A).

In summary, the rice PRI showed specificity of the proximal promoter region over the distal promoter region, intergenic regions and CDS, but mixing of a positive signal from the 5' UTR, which was not observed in *Arabidopsis*, produced some reduction in the specificity.

The threshold of the PRI for the prediction of TSS in rice was examined and a value of 0.06 was selected for further studies into TSS prediction (Supplemental Figure S5).

### Evaluation of promoter context of genic and non-genic promoters

**Figure 7:**
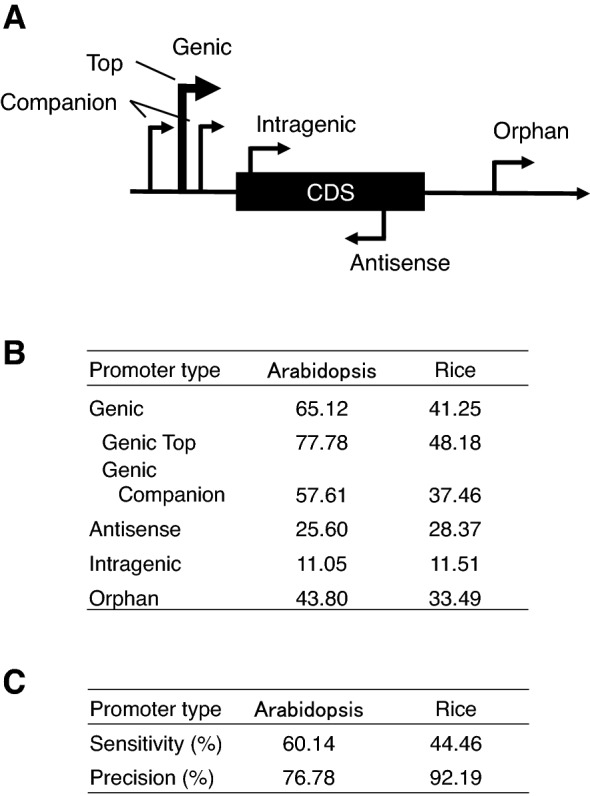
Prediction scores for various promoter types. (**A**) Illustration of promoter types. Experimentally identified TSS clusters (= promoters) are classified as shown (Tokizawa et al., 2017). There are three genic promoters for a gene on average. The strongest one is called the Genic Top, and the others Genic Companions. Orphans are the promoters whose corresponding gene model does not exist. (**B**) Sensitivity of prediction according to promoter types. Genome-wide prediction is done. (**C**) Results of promoter prediction considering promoter direction for Genic Top promoters. In this prediction, results are negative if the prediction of the direction is wrong, even if positional prediction is correct. Directional prediction was achieved by comparison of the peak height for the forward and reverse directions.

Figure 7A illustrates the types of promoters, classified according to their relative position and direction to gene structure. We previously detected around three genic promoters per protein-coding gene. The most active promoter among them is referred to as the Genic Top promoter and the other two as Genic Companion promoters^3^. Gene expression is primarily achieved by the Genic Top promoters, and contribution of the Genic Companion promoters is negligible, in general. In addition to these genic promoters, Intragenic and Antisense promoters were also commonly detected. Orphan promoters are those which are not connected to any gene models.

Our PRI system evaluates middle-range promoter context of a region from − 750 to − 60 excluding the core promoter region. Therefore, promoters lacking the middle-range promoter context are expected to give low scores and fail in the TSS prediction.

We then evaluated the prediction sensitivity for each promoter category in *Arabidopsis* and rice. Because the prediction methodology was aimed at the Genic Top promoters, this category was expected to produce the best results, but we investigated whether the other categories were detectable.

The Genic Top promoter gave highest scores for detection sensitivity in both *Arabidopsis* (77.78%) and rice (48.18%) (Fig. 7B). In both *Arabidopsis* and rice, the scores of the Genic Companion promoters were slightly lower than those of the Genic Top promoters, suggesting that the former promoters are less mature than the latter ones. The score of the rice promoters (48.18%) was considerably lower than that of the *Arabidopsis* promoters (77.78%), reflecting the reduced specificity of PRI in rice from that in *Arabidopsis* (Figs. 2 & 6). Compared with these genic promoters, the scores of the Antisense and Intragenic promoters were markedly reduced. These results suggest that these promoters lack the middle-range promoter context, and have only the local-range context around TSS. These two types of promoters often overlap with CDS.

Orphan promoters, which are not connected to any gene models, and which produce lower levels of expression than Genic promoters^3^, produced scores lower than Genic Companion and higher than Antisense in both *Arabidopsis* and rice. These results suggest that Orphan promoters have the promoter context, but it is weak and immature.

Figure 7C shows the scores for sensitivity and precision of prediction, including that of promoter detection for Genic Top promoters. The inclusion of directional prediction only slightly reduced the prediction scores, revealing accurate prediction of promoter direction. The distribution profiles of PRI for Genic Companion, Orphan, Intragenic, and Antisense promoters are shown in Supplemental Figure S6. Only Genic Companion promoters produced a peak in the proximal promoter region with correct directional difference. Orphan promoters did produce a peak in the appropriate region or the correct directional difference of the scores, but these features are subtle and height of its weak peak was below zero. Intragenic and Antisense promoters did not have a recognizable peak in the proximal region, again demonstrating a complete lack of the middle-range promoter context.

### Improvement of specificity of PRI in rice

The rice PRI produced a positive score in the 5' UTR in addition to the proximal promoter region (− 200 to − 60) (Fig. 6), which is thought to give a lower score of the Genic Top promoters of rice than of *Arabidopsis*. Thus we developed the 5' UTR Index (FUI) in order to improve the specific detection of rice promoter regions. The FUI was calculated as for PRI by subtracting IGI750_450, with an exchange of the frequency of the proximal promoter region to that of the 5' UTR. The high specificity of FUI for the 5' UTR over other genic regions is shown in Supplemental Figure S7A. The FUI scores along the promoter region are shown in Panel B. The threshold of FUI in the rice genome is shown in Supplemental Figure S7, and 0 (zero) was selected as the threshold (Panel C).

With the aid of the FUI, we set up a modified scheme for TSS prediction (Supplemental Figure S8A). The TSS, 5' UTR, and CDS should be located in this order, and our initial plan was to restrict search to this order to improve the accuracy of TSS prediction using the FUI, as shown in Supplemental Figure S8A. This scheme requires the presence of a 5'UTR region around the predicted TSS, excluding predicted TSSs located directly upstream of the CDS without a 5' UTR signal in its vicinity.

The results of the TSS prediction produced using the previous method and the modified scheme combining the PRI and FUI are shown in Panels B and C, respectively. These graphs are summarized according to the expression level of the promoters. The modified method produced higher precision regardless of the expression level, but lower sensitivity. As a result, the F value became comparable. From these results we concluded that the modified method using FUI did not improve TSS prediction in rice.

### Comparison with TSSPlant

We compared our PRI-based TSS prediction with that produced by TSSPlant^13^. This is a method involving the evaluation of a shorter range than ours, from − 200 to + 51 compared to our − 750 to − 60. TSSPlant evaluates the proximal and local-range sequences around the TSS plus some extensions, and the PRI uses middle-range promoter context, excluding these short-range TSS contexts. Supplemental Figure S9 demonstrates the results of TSSPlant for the detection of positive and negative Arabidopsis sequences from − 250 to + 51, which had 94% sensitivity and 69.12% precision, comparable to previously reported results^13^. For comparison with PRI-based prediction, 2 kb sequences containing TSS (Genic Top) were subjected to PRI and TSSPlant for prediction of the position of the TSS. Because TSSPlant does not have any schemes to select the best TSS, we ran two strategies: acceptance of all the predictions, and selection of one TSS with the highest score within the 2 kb region. Both results are included in the table.

As shown in Table 1, PRI-based prediction gave much higher F values than TSSPlant for both Arabidopsis and rice. These results demonstrate the superiority of the PRI-based prediction over TSSPlant. One possible cause of the results is the superiority of the evaluation of middle-range promoter sequences over short-range evaluation. However, these two strategies are not mutually exclusive, but can be integrated based on the differences in their scoped ranges.

**Table 1:** Comparison with TSSPlant.

	Number of predicted promoters	TP	Sensitivity (%)	Precision (%)	F (%)
**Arabidopsis**					
Promoter index	94	67	67.00	71.28	69.07
**TSSPlant**					
No-selection	402	69	69.00	17.16	27.49
Max score	100	36	36.00	36.00	36.00
**Rice**					
Promoter index	61	33	33.00	54.10	40.99
**TSSPlant**					
no-selection	416	56	56.00	13.46	21.71
max score	100	15	15.00	15.00	15.00

Two kilobase sequences from CDS of 100 randomly selected genes of Arabidopsis and rice were subjected to our prediction (Promoter Index) and TSSPlant. *TP* true positive, *F* harmonic mean of sensitivity and precision. Success of the prediction by TSSPlant indicates the presence of a Genic Top TSS within 100 from the predicted TSS point.

### Sequences affecting the PRI score

**Figure 8:**
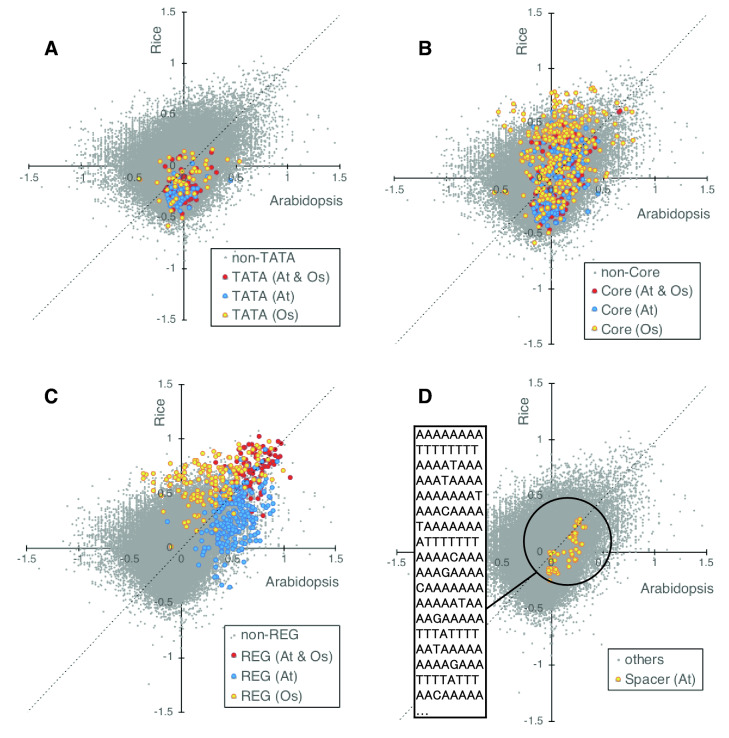
PRI score of promoter constituents in *Arabidopsis* and rice. Horizontal and vertical axes are PRI of *Arabidopsis* and rice, respectively. All the octamer sequences are presented in gray dots, and highlighted with color for several categories of promoter constituents: (**A**) TATA, (**B**) Core elements (TATA, Y Patch, GA, and CA), (**C**) REG (Regulatory Element Group), (**D**) Spacer sequences in Arabidopsis which are the most frequently occurring octamers in the promoter region excluding functional elements (REG and Core).

Lastly, we examined which promoter-constituting sequences contributed to the high PRI score. The scatter plots in the four panels of Fig. 8 are all the same, and show the PRI scores of all the octamer sequences in Arabidopsis and rice. The plot indicate moderate or low conservation between Arabidopsis and rice PRI scores. Degree of the conservation was examined by cross application of the PRI scores to rice and Arabidopsis promoter sequences. As shown in Supplemental Figure S11, application of the Arabidopsis PRI score to rice promoter sequence (− 200 to − 60) gave a lower score than to the Arabidopsis sequences, but still positive value was obtained, so the Arabidopsis PRI table is applicable to rice sequences. Application of the rice PRI score to Arabidopsis promoter sequences (− 200 to − 600) resulted in a negative value, therefore the rice PRI table is not applicable to Arabidopsis sequences.

Each panel highlights different octamer groups. Octamers of the TATA box are shown in Panel A, and this group does not contribute to high PRI in either *Arabidopsis* or rice.

Panel B shows all the core elements including TATA, Y Patch, GA, and CA^12^. This group is a mixture of varied scores, both positive and negative, and does not show a tendency toward high or low PRI scores. We concluded that these two groups do not produce high PRI scores as a whole. The results shown in Panels A and B are consistent with core-type independent prediction by PRI (Fig. 5A).

Panel C shows the REGs. The REG octamers have some differentiation between Arabidopsis (218 octamers) and rice (152), with considerable conservation (90)^12^. The conserved REG octamers gave high PRI scores in both *Arabidopsis* and rice. *Arabidopsis*-specific REG octamers tended to have high PRI scores only in *Arabidopsis*, and rice-specific REGs showed high PRI scores only in rice. These results indicate that REGs gave high PRI scores in both *Arabidopsis* and rice, and suggest that loss of REGs results in decline in the PRI score, consistent with the results in Fig. 5D.

Panel D emphasizes the spacer sequences paving the *Arabidopsis* promoter region, which are the most frequently detected 50 octamers in the promoter region, excluding core and REG (sum of 200_60 & 750_450). This group has PRI scores varying from high to low in *Arabidopsis*, revealing that not all the highly detected sequences in the promoter region had a preference toward the proximal promoter region. Comparison with the rice scores revealed that the high and low scores of octamers in this group were well conserved between the two species. These results suggest that sequences paving the promoter context are conserved to some extent, despite the difference of GC content between the two (Arabidopsis = 36%, rice = 43%^14^).

In summary, our analyses revealed that REG and a part of the spacer sequences contribute to high PRI scores, and core elements, especially the TATA box, do not. The PRI method was developed to detect the middle-range promoter context by evaluation of differential appearance within a region from − 750 to − 60 relative to TSS. Therefore, these results, positive effects of REG and spacers and negligible effects of core elements, are reasonable. These observations provide the basis for core-type-independent promoter detection using PRI.

## Discussion

Decades ago, prediction of the promoter regions in genome sequences was attempted by finding the TATA box, the first core promoter element identified in a wide range of eukaryotes, and the CpG islands, which are known to be accompanied by mammalian promoters. This core element-based approach alone turned out to be insufficient for prediction^15^. Later genome-wide promoter analyses revealed that a considerable number of promoters do not have either a TATA box or CpG islands^2^, which means that these coreless promoters cannot be detected using the core element-based promoter search. In addition, the existence of coreless promoters implies that the known core elements are not indispensable for transcriptional initiation. Higher plants do not have the CpG type^4^ and many promoters do not have any detectable core promoter elements^9^, so core-based promoter prediction could be less useful in plants.

Another approach to promoter detection utilizes the structural features of the DNA, accompanied by DNA sequences^15,16^. These features are applicable to mammals and fish but not to yeast, insects, or higher plants^16^. Therefore, the features established in mammals are not a general rule among eukaryotes.

Recently, machine learning has been introduced into promoter prediction. Methodologically, this is a powerful general-purpose strategy which is applicable to a wide range of problems, including image recognition and speech interpretation. It requires positive and negative sequences for learning, and the quality of the output depends on the number, diversity, and range of the sequences utilized. This approach has had some success^13^, but improvement of the methodology is still required for better prediction. One big problem with the machine learning strategy is that it does not necessarily advance our knowledge, even if the prediction system works well.

The methodologies mentioned above can be combined, and an integrated strategy has recently been reported^17^. Combinational approaches are expected to produce better prediction accuracy than stand-alone methods, but if essential knowledge is lacking, combination does not cover this lack.

Our initial plan for quantification of the promoter context in a manner independent of the core promoter types was to focus on the spacer sequences in the promoter region. After development, one thing we did not expect was the positive contribution of REG to our evaluation method. Even so, evaluation of the promoter context without taking account of the core elements has been successfully established in this study. This is the first report of middle-range (~ 1 kb) promoter evaluation method solely based on the nucleotide sequence. Pinpoint prediction of TSS, which is local-range prediction, should be achieved using other methodologies, such as YR Rule^9,12^ and TSSPlant^13^.

The requirements for the spacer sequences in the promoter region are not well understood. We assume that they include the absence of undesirable functions and the tuning of nucleosome positioning. Strand-specific differences in the PRI scores (Figs. 3, 6D) and precise prediction of the transcriptional direction (77% to 92% precision, Fig. 7C) suggest that there is another requirement for the spacer sequence, which facilitates the determination of transcriptional direction. The molecular mechanisms behind them remain to be elucidated.

A longstanding question is the length of the actual promoter of the Genic Top type. The least strict determination of the promoter end is the closest edge of the adjacent gene. The distance to the neighboring gene from a TSS on average in the packed *Arabidopsis* genome is between around 1800 bp (head-to-head junction of the TATA type) and around 500 bp (tail-to-head junction of the Coreless type)^18^. The actual length of *Arabidopsis* promoters should be less than this.

The PRI starts rising from around − 500 bp toward the downstream region (*e.g.,* Fig. 3A). Therefore, the region from − 500 to the TSS, which has high scores for the PRI, is a possible promoter region in *Arabidopsis* with respect to the promoter context. Our previous studies of *Arabidopsis* 80 accessions detected pressure for sequence conservation starting from − 500 or − 400 toward the TSS (Fig. 8,^3^). Assuming that the promoter region is more conserved than the intergenic region, this observation suggests that the promoter region starts from − 500 or − 400 toward the downstream region.

Together, these results consistently suggest that the *Arabidopsis* promoter region is from − 500 to the TSS. This is a general trend, and does not exclude the presence of exceptional promoters with an extended region, so caution is necessary when analyzing an individual promoter.

Non-genic promoters—Orphan, Intragenic, and Antisense—had much shorter regions of high PRI than the genic types. Supplemental Figure S6 shows the trend in PRI, and Figure S15^3^ shows the sequence conservation of Orphan promoters, suggesting that they lack or have considerably shorter promoter contexts than Genic Top promoters.

One important feature of the PRI is its positive relationship with the gene expression level (Fig. 5B). Because the PRI does not evaluate the transcribed region, the index does not reflect the stability of the transcripts, but only reflects transcriptional activity. On the assumption that active promoters are more mature than non-active ones, we suggest that the PRI reflects the maturation state of each promoter.

Comparative analyses between *Arabidopsis* and rice revealed that the PRI of rice was less specific than that of *Arabidopsis*. The major reason is cross-talk of the 5' UTR to the index in rice, due to their sequence similarity (Fig. S7A), which was not observed in *Arabidopsis* (Fig. 2C). Differences between *Arabidopsis* and rice with respect to sequence preference in the 5' UTR would be reflected by genomic differences in the GC content, which is higher in rice^14^.

A comparison of the score between frequently observed non-core sequences in *Arabidopsis* and rice promoters, which we consider typical spacer sequences, detected a conservative trend in the score (Fig. 8D). This observation suggests that preferred spacer sequences in *Arabidopsis* and rice promoters are generally conserved, despite the difference in the GC content of these genomes. This finding suggests that there is a positive function of the spacer sequence in addition to its neutral, or non-disturbing effects on transcriptional initiation.

For the prediction of genic TSSs, the utilization of information about the position of the CDS considerably elevated the prediction scores (Fig. 4). This elevation may suggest that downstream CDS is also a factor determining the promoter context, in addition to the DNA sequence in the promoter region (− 1000 to − 50). This idea is not supported by our knowledge of the molecular mechanisms of transcriptional initiation^6^. However, our recent report on TSS generation and selection in Arabidopsis revealed that the insertion of foreign CDS generated new TSS in the 5' proximity of the inserted CDS, and that when a core promoter was triplicated between CDS and the regulatory region, the closest core to the CDS provided the most frequent TSSs^19^, suggesting that the downstream CDS stimulated TSSs in the closest core. Our results of the elevation of TSS prediction by utilization of CDS information support the idea presented by Kudo et al*.*, and suggest that this is a genome-wide phenomenon. This phenomenon can be explained in part by enhancement of the transcript stability by a short 5' UTR, or by transcriptional stimulation by a CDS of the near upstream region, with a completely unknown mechanism.

## Methods

### Data source

TSS-seq data from several TSS libraries of *Arabidopsis thaliana* and rice was prepared previously^3^ (Tokizawa M., Kusunoki, K., Ushijima, T., Matsushita, T., Kanesaki, Y., Suzuki, Y., Koyama, H., Yamamoto, Y.Y., unpublished results). For Arabidopsis analysis, 324,461 TSS clusters, including 21,673 Genic Top clusters, were utilized. For rice, 250,548 TSS clusters, including 22,405 Genic Top clusters, were used. TAIR10^20^ and RGAP7^21^ were used as genome annotation information and the genome sequence for *Arabidopsis* and rice, respectively. Promoter sequences were extracted from the genome sequences based on the position of the peak TSS of TSS clusters. Other *Arabidopsis* and rice sequences were obtained from TAIR10 and RGAP7, respectively. Octamer sequences for promoter elements (core, TATA, and REG) of Arabidopsis and rice were determined in our previous reports^9,12,22^.

### Data process

Sequence analysis was achieved using home-made Python, Perl and shell scripts and summarized using Excel (Microsoft Japan, Tokyo). Our PRI tables of Arabidopsis and rice prepared with all the chromosomes for training, and scripts for preparation of PRI tables and for sequence evaluation with the tables are available at GitHub (https://github.com/yyyamamoto/TssPrediction). The preparation of the PRI tables for *Arabidopsis* and rice are described in Supplemental Fig. 1. The scoring of genomic sequences with PRI/IGI/FUI tables were done using Chrom_scan.py, and peak picking of the scanned data after smoothing with a bin of 151 bp using peak_find_SG.py. TSSPlant for Linux^13^ (https://www.cbrc.kaust.edu.sa/download/) was locally run with default settings.

*Arabidopsis* spacer sequences in the promoter region, used in Fig. 8D, were selected as the most highly observed octamers from − 750 to − 450 and − 200 to − 60 of *Arabidopsis* Genic Top promoters.

Finalized flow of the methods are illustrated in Supplemental Figure S12.

## Supplementary Information

Supplementary Information.

## References

[1] Suzuki Y (2001). Identification and characterization of the potential promoter regions of 1031 kinds of human genes. Genome Res..

[2] Carninci P (2006). Genome-wide analysis of mammalian promoter architecture and evolution. Nat. Genet..

[3] Tokizawa M (2017). Identification of Arabidopsis genic and non-genic promoters by paired-end sequencing of TSS tags. Plant J..

[4] Yamamoto YY (2007). Differentiation of core promoter architecture between plants and mammals revealed by LDSS analysis. Nucleic Acids Res..

[5] Frith MC (2008). A code for transcription initiation in mammalian genomes. Genome Res..

[6] Carey MF, Peterson CL, Smale ST (2009). Transcriptional Regulation in Eukaryotes.

[7] Smale ST, Kadonaga JT (2003). The RNA polymerase II core promoter. Annu. Rev. Biochem..

[8] Bajic VB (2006). Mice and men: Their promoter properties. PLoS Genet..

[9] Yamamoto YY (2009). Heterogeneity of Arabidopsis core promoters revealed by high-density TSS analysis. Plant J..

[10] Morton T (2014). Paired-end analysis of transcription start sites in Arabidopsis reveals plant-specific promoter signatures. Plant Cell.

[11] Mejia-Guerra MK (2015). Core promoter plasticity between maize tissues and genotypes contrasts with predominance of sharp transcription initiation sites. Plant Cell.

[12] Yamamoto YY (2007). Identification of plant promoter constituents by analysis of local distribution of short sequences. BMC Genom..

[13] Shahmuradov IA, Umarov RK, Solovyev VV (2017). TSSPlant: A new tool for prediction of plant Pol II promoters. Nucleic Acids Res..

[14] Yu J (2002). A draft sequence of the rice genome (Oryza sativa L. ssp. indica). Science.

[15] Bajic VB, Tan SL, Suzuki Y, Sugano S (2004). Promoter prediction analysis on the whole human genome. Nat. Biotechnol..

[16] Abeel T, Saeys Y, Bonnet E, Rouze P, Van de Peer Y (2008). Generic eukaryotic core promoter prediction using structural features of DNA. Genome Res..

[17] de Medeiros Oliveira M, Bonadio I, Lie de Melo A, Mendes Souza G, Durham AM (2021). TSSFinder-fast and accurate ab initio prediction of the core promoter in eukaryotic genomes. Brief Bioinform..

[18] Yamamoto YY, Yoshioka Y, Hyakumachi M, Obokata J (2011). Characterization of core promoter types with respect to gene structure and expression in *Arabidopsis thaliana*. DNA Res..

[19] Kudo H (2021). Cryptic promoter activation occurs by at least two different mechanisms in the Arabidopsis genome. Plant J..

[20] Lamesch P (2012). The Arabidopsis information resource (TAIR): Improved gene annotation and new tools. Nucleic Acids Res..

[21] Kawahara Y (2013). Improvement of the Oryza sativa Nipponbare reference genome using next generation sequence and optical map data. Rice.

[22] Hieno A (2014). ppdb: Plant promoter database version 3.0. Nucleic Acids Res..

