# Supplementary material for: Sequence-based evaluation of promoter context for prediction of transcription start sites in Arabidopsis and rice
Source: Sci Rep. 2022 Apr 28;12:6976. doi: 10.1038/s41598-022-11169-w (PMC9050755; doi:10.1038/s41598-022-11169-w)
Supplement: Supplementary file 1 — Supplementary Information. [file 41598_2022_11169_MOESM1_ESM.pdf]

1. Counting occurrence of octamers

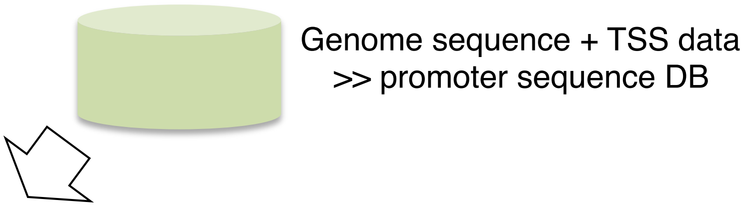

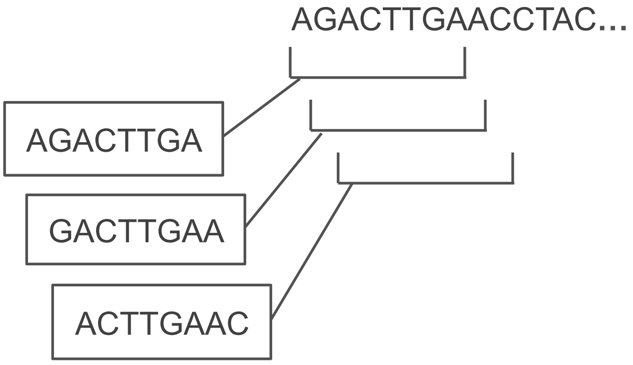

Octamer	Occurrence
AAAAAAAA	5144
AAAAAAAT	1342
⋮	
CCCCCCCG	76
CCCCCCCC	43

2. Conversion to score

$$
\begin{cases}
\text{when count} > 0: & \text{score} = \log_{10} \dfrac{\text{occurrence of an octamer}}{\text{occurrence of all the octamers}} - \log_{10} \dfrac{1}{4^8} \\
\text{when count} = 0: & \text{score} = 0
\end{cases}
$$

Intergenic Index ("IGI")

- IGI200_60: score made from region from -200 to -60 bp
- IGI750_450: score made from region from -750 to -450 bp

Promoter Index ("PRI")

- PRI200_60-750_450: IGI200_60 – IGI750-450

Octamer	Score
AAAAAAAA	1.96
AAAAAAAT	1.44
⋮	
CCCCCCCG	-1.74
CCCCCCCC	-0.27

Supplemental Figure S1. Preparation of Promoter Index
Each index table is a set of all the octamer sequences ($4^8 = 65{,}536$). **1.** A specific region in the promoter sequences of a genome is cut out and subjected to octamer counting. **2.** Count is converted into score as shown in the figure, which is log expression of relative occurrance normalized with random occurrance. An octamer with unbiased occurrance gives a score of zero. Subtraction of two IGI gives score of preferential occurrance in the region of the subtracted IGI over the subtracting IGI. In the case of shown PRI, a positive score means preferential occurrance in the proximal region.

	Area width	TP ratio (%)	Rank
PRI200_60-800_200	600	52	7
PRI200_60-800_350	450	55	3
PRI200_60-750_300	450	54	5
PRI200_60-700_250	450	51	8
PRI200_60-650_200	450	46	10
PRI200_60-800_500	300	56	2
* PRI200_60-750_450	300	58	1
PRI200_60-700_400	300	55	3
PRI200_60-650_350	300	51	8
PRI200_60-600_300	300	53	6
PRI200_60-550_250	300	42	11
PRI200_60-500_200	300	42	11

Supplemental Figure S2. Selection of promoter region for preparation of promoter index
Promoter index is calculated by subtraction of a distal region index from the proximal region index (-200 to -60 bp from TSS). The table shows results of TSS prediction of randomly selected 100 protein-coding genes of Arabidopsis Chr 1. Area width: width of the proximal region. TP: true positive. Rank: order of the index from the one giving the highest TP. A promoter index giving the highest TP, which is Rank 1, is highlighted with an asterisk.

Threshold		Number of predicted promoters	TP	Sensitivity (%)	Precision (%)	F (%)
*	0	21397	14672	67.76 (0.96)	68.67 (1.07)	68.21 (1.01)
	0.0001	21391	14669	67.74 (0.94)	68.67 (1.03)	68.20 (0.99)
	0.0002	21387	14664	67.72 (0.94)	68.66 (1.03)	68.18 (0.99)
	0.0003	21384	14665	67.72 (0.93)	68.67 (1.01)	68.19 (0.97)
	0.001	21364	14649	67.66 (1.02)	68.68 (1.12)	68.16 (1.06)
	0.002	21337	14629	67.57 (1.00)	68.67 (1.10)	68.11 (1.05)
	0.003	21310	14614	67.52 (0.98)	68.71 (1.11)	68.11 (1.04)
	0.01	21027	14299	66.08 (1.00)	68.15 (1.13)	67.10 (1.05)
	0.02	20424	13494	62.35 (1.12)	66.19 (1.07)	64.21 (1.08)
	0.03	19355	12219	56.38 (0.87)	63.12 (0.77)	59.56 (0.82)
	0.1	3379	2114	9.75 (0.32)	62.69 (1.29)	16.88 (0.47)
	0.2	44	19	0.09 (0.03)	43.26 (17.68)	0.17 (0.07)
	0.3	12	4	0.02 (0.02)	28.33 (26.67)	0.04 (0.04)

Supplemental Figure S3. Selection of threshold of PRI

Results of prediction with various threshold (Fig. 4) are shown. Genome-wide prediction for protein-coding genes is done. TP: true positive. Sensitivity: TP/ positives. Precision: TP/ predictions. F: harmonic mean of Sensitivity and Precision. A threshold giving the highest F value is highlighted with an asterisk. Each chromosome was subjected to prediction with a PRI table prepared with the rest 4 chromosome data. Average and standard deviation of 5 chromosome data are shown.

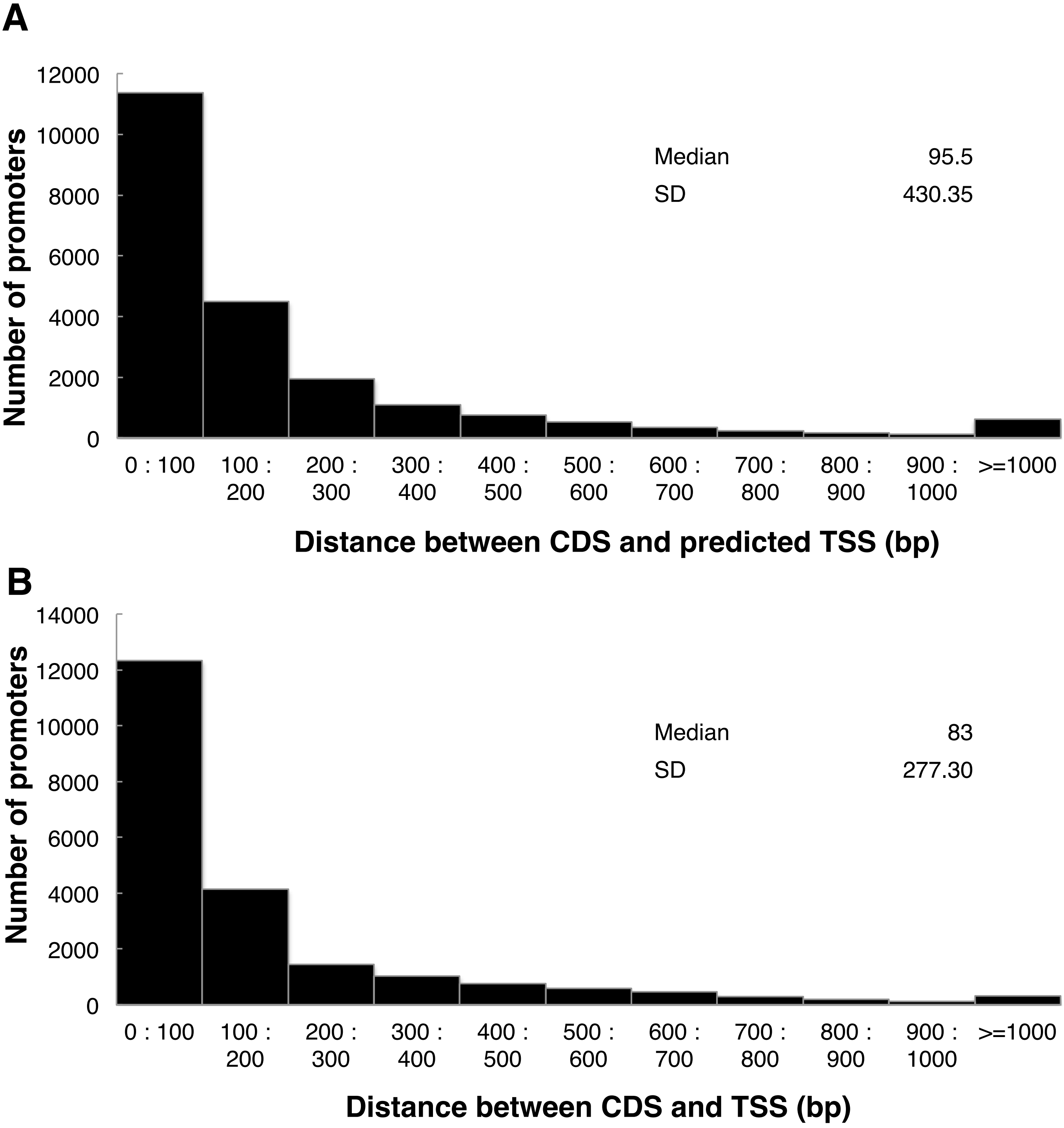

Supplemental Figure S4. Predicted and actual distance between CDS and TSS
Data of Arabidopsis Genic Top Promoters is shown. 0:100 means 0 to 99 bp and 100 bp is excluded from the fraction. (**A**) Distance from CDS to predicted TSS. Middle of the predicted TSS area is used to determine the distance. (**B**) Actual distance from CDS to actual TSS, which is the peak TSS in a Genic Top TSS cluster. TSS data is obtained from TSS-Seq analysis (Tokizawa *et al*., 2017).

Threshold	Number of predicted promoters	TP	Sensitivity (%)	Precision (%)	F (%)
0	18267	8069	38.10 (2.80)	44.54 (2.80)	41.07 (2.81)
0.001	18179	8064	38.09 (2.79)	44.72 (2.78)	41.13 (2.80)
0.002	18088	8055	38.03 (2.80)	44.88 (2.79)	41.17 (2.81)
0.003	18008	8053	38.02 (2.77)	45.05 (2.72)	41.23 (2.76)
0.004	17924	8056	38.03 (2.77)	45.27 (2.69)	41.33 (2.75)
0.005	17828	8051	38.01 (2.75)	45.49 (2.69)	41.41 (2.74)
0.006	17734	8039	37.95 (2.74)	45.67 (2.70)	41.45 (2.74)
0.007	17661	8023	37.86 (2.72)	45.74 (2.66)	41.43 (2.72)
0.008	17575	8017	37.81 (2.69)	45.91 (2.67)	41.47 (2.70)
0.009	17504	8002	37.74 (2.70)	46.01 (2.67)	41.46 (2.71)
0.01	17428	7994	37.70 (2.71)	46.15 (2.62)	41.49 (2.70)
0.01	17428	7994	37.70 (2.71)	46.15 (2.62)	41.49 (2.70)
0.02	16549	7857	37.05 (2.74)	47.67 (2.53)	41.69 (2.69)
0.03	15723	7754	36.54 (2.75)	49.48 (2.48)	42.02 (2.70)
0.04	14907	7599	35.76 (2.82)	51.03 (2.57)	42.04 (2.80)
0.05	14067	7418	34.85 (2.88)	52.75 (2.71)	41.96 (2.93)
* 0.06	13331	7284	34.24 (2.84)	54.63 (2.62)	42.08 (2.89)
0.07	12614	7059	33.21 (2.77)	56.02 (2.76)	41.68 (2.90)
0.08	11907	6838	32.16 (2.65)	57.43 (2.38)	41.21 (2.76)
0.09	11277	6650	31.34 (2.66)	59.02 (2.37)	40.91 (2.81)
0.1	10647	6387	30.07 (2.40)	60.07 (2.05)	40.05 (2.57)

Supplemental Figure S5. Determination of threshold for rice TSS prediction

Average and standard deviation of 6 prediction sets for Chr 1-2, 3-4, 5-6, 7-8, 9-10, and 11-12 are shown. For each prediction set, a PRI table was prepared with TSS data excluding data of the two chromosomes subjected to prediction. TP: true positive, Sensitivity: TP/ positives, Precision: TP/ predictions, F: harmonic mean of Sensitivity and Precision. A threshold giving the highest F value is highlighted with an asterisk.

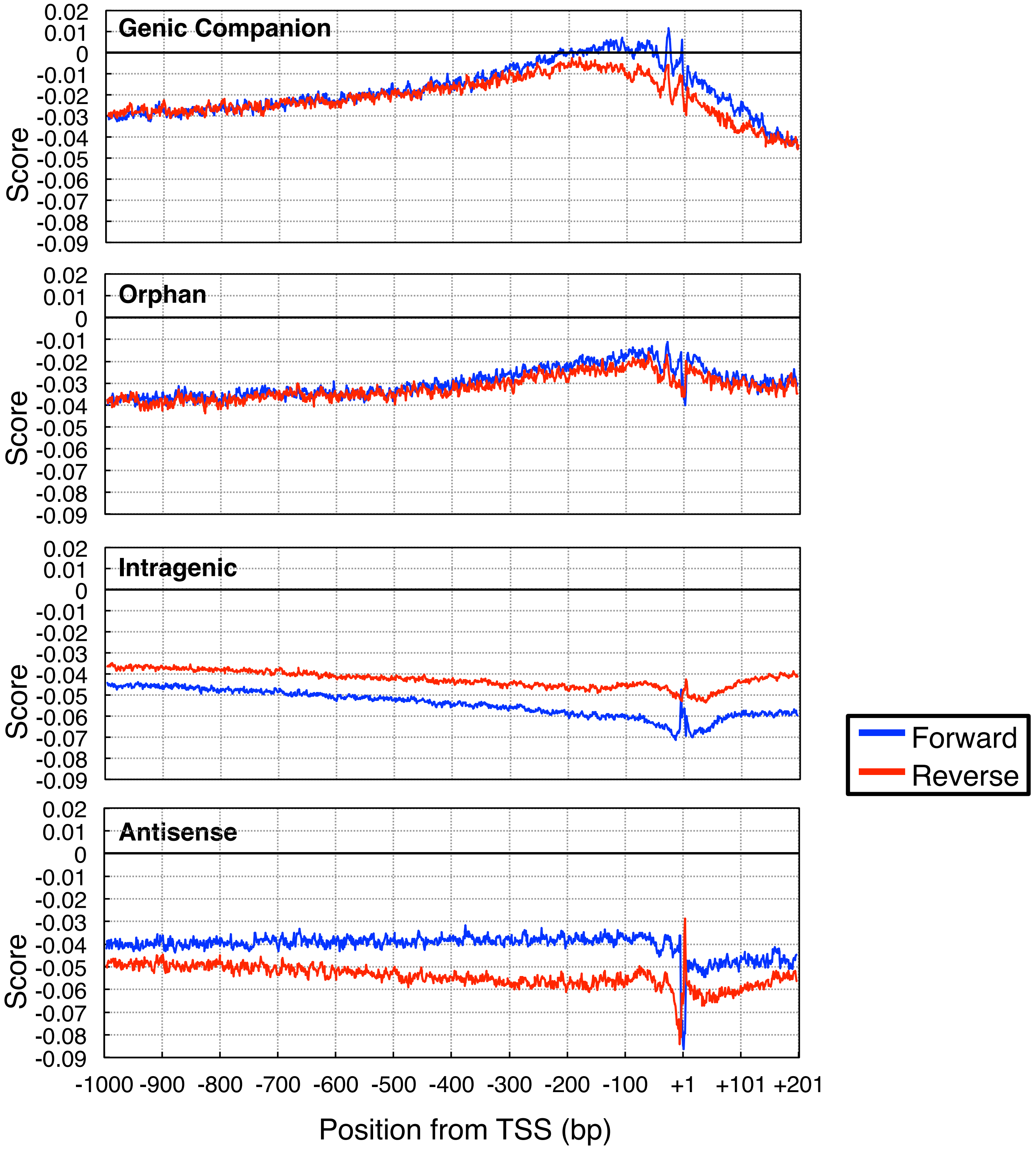

Supplemental Figure S6. PRI distribution of promoter types in Arabidopsis
Average of the genome-wide data is shown. Vertical axis shows PRI score.

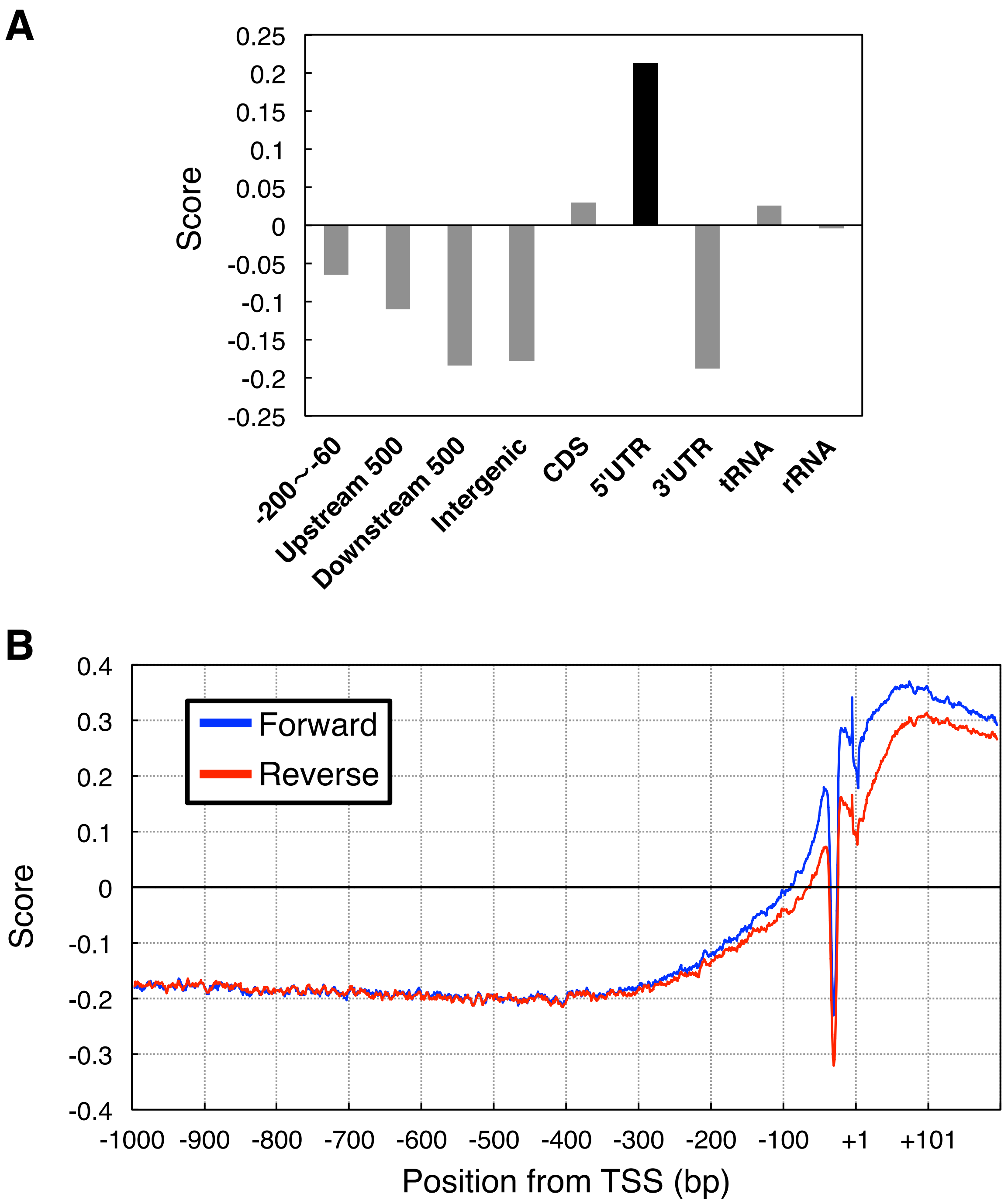

Supplemental Figure S7. Preparation of 5'UTR Index (FUI) in rice (continue)

C

Threshold	Number of predicted promoters	TP	Sensitivity (%)	Precision (%)	F (%)
* 0	11180	7429	35.00 (2.64)	66.50 (2.17)	45.84 (2.76)
0.001	11175	7426	35.00 (2.64)	66.50 (2.15)	45.83 (2.75)
0.002	11170	7423	34.99 (2.66)	66.52 (2.18)	45.83 (2.77)
0.003	11159	7420	34.97 (2.65)	66.55 (2.11)	45.82 (2.75)
0.01	11088	7399	34.85 (2.61)	66.78 (1.85)	45.77 (2.69)
0.02	10882	7323	34.48 (2.64)	67.26 (1.78)	45.55 (2.71)
0.03	10676	7275	34.26 (2.66)	68.05 (1.66)	45.53 (2.73)
0.1	8238	6065	28.58 (2.30)	73.61 (1.32)	41.13 (2.58)
0.2	3731	2861	13.44 (1.56)	76.33 (1.80)	22.82 (2.31)
0.3	471	344	1.61 (0.28)	72.75 (9.17)	3.15 (0.55)

Supplemental Figure S7. Preparation of 5'UTR Index (FUI) in rice
(**A**) Rice 21,645 5'UTR sequences were subjected to FUI as illustrated in Supplemental Figure 1 (IGI), and the preprared index table was applied to the genic regions and transcribled region of a few gene types as shown. Average of genome-wide analysis is shown. (**B**) Distribution of FUI around the promoter region. Average of genome-wide data is shown. (**C**) Determination of threshold of PRI for the FUI-aided prediction. Genic Top promoters were subjected to the prediction.

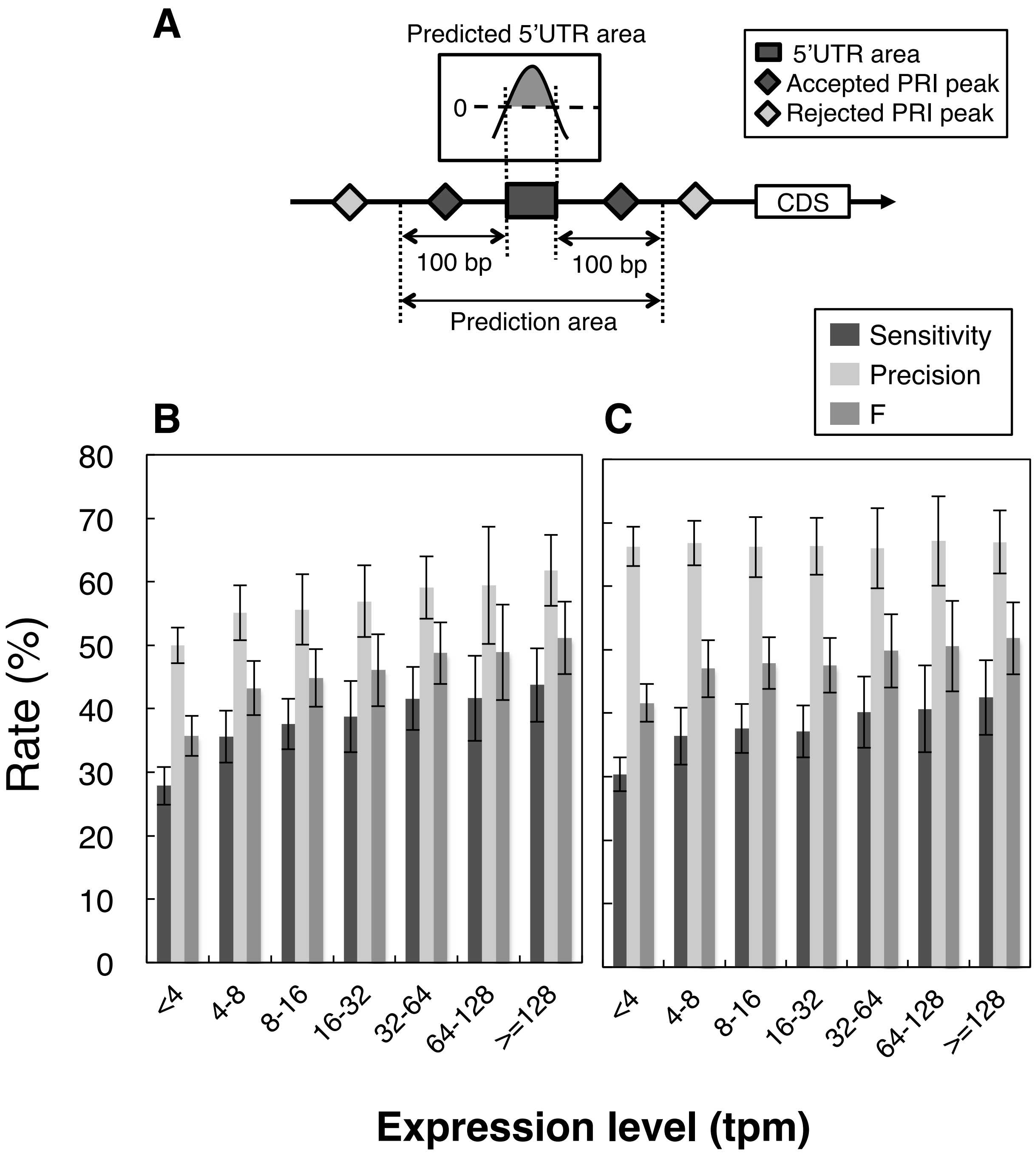

Supplemental Figure S8. **FUI-assisted TSS prediction in rice**
(**A**) Illustration of FUI-assisted prediction. In addition to the standard TSS prediction for protein-coding genes, presence of FUI-positive area is required near the predicted TSS. In this way, Rejected PRI peaks in the illustration (rhombuses in light gray) are excluded from the prediction. (**B**) Prediction scores with PRI according to expression level. (**C**) Prediction scores with FUI according to expression level.

	TP	FN	TN	FP	Sensitivity (%)	Precision (%)	F (%)
TSSPlant	94	6	58	42	94.00	69.12	79.66

Supplemental Figure S9. Application of TSSPlant for Arabidopsis TSS prediction

Randomly selected 100 sequences from -200 to +51 bp of Arabidopsis protein coding genes relative to the peak of Genic Top TSS clusters (positive sequences) plus randomly selected 100 gene fragments 251 bp long starting from the head of the second exon (negative sequences) were subjected to TSSPlant. Success of the prediction means presence of peak of Genic Top TSS cluster (Tokizawa et al., 2017) within 100 bp from the predicted TSS point. TP: ture positive, FN: false negative, TN: true negative, FP: false positive, F: harmonic mean of Sensitivity and Precision. Figure at columns of TP, FN, TN, and FP means number of corresponding sequences.

AAAAAAAA	ATTTTTTT
AAAAAAAT	AGAAAAAA
AAAAAAAG	ACAAAAAA
AAAAAAAC	TAAAAAAA
AAAAAATA	TATTTTTT
AAAAAAGA	TTAAAAAA
AAAAAACA	TTATTTTT
AAAAATAA	TTTAAAAA
AAAAATAT	TTTATTTT
AAAAAGAA	TTTTAAAA
AAAAACAA	TTTTATTT
AAAATAAA	TTTTTATT
AAAAGAAA	TTTTTTTA
AAAACAAA	TTTTTTTT
AAATAAAA	TTTTTTTG
AAATAAAT	TTTTTTTC
AAATTAAA	TTTTTGTT
AAAGAAAA	TTTTTCTT
AAACAAAA	TTTTGTTT
AATAAAAA	TTTTCTTT
AATAAAAT	TTTGTTTT
AATTAAAA	TTTCTTTT
AAGAAAAA	GAAAAAAA
AACAAAAA	CAAAAAAA
ATAAAAAA	CTTTTTTT

Supplemental Figure S10. The most frequently observed Arabidopsis promoter sequences
The shown 50 octamers represent Arabdiopsis spacer sequenes in the promoter region used in Figure 8D.

		PRI Score	
		**Arabidopsis**	**rice**
**Evaluated promoter sequence** (-200 ~ -60)	**Arabidopsis**	0.022	-0.055
	**rice**	0.0080	0.048

Supplemental Figure S11. Cross-application of PRI score table

PRI scores prepared from Arabidopsis and rice training data were subjected to evaluation of Arabidopsis and rice promoter sequences from -200 to -60 bp relative to TSS. Evaluated sequences were prepared based on our TSS info (Genic Top of Arabidopsis (Tokizawa *et al*., 2017) and rice (Tokizawa *et al*., unpublished)).

Preparation of PRI table

Promoter sequences (-1,000 to -1 relative to Peak position of Top Genic TSS cluster)

⇩ TSS_count.py + 8bp_all.txt

Occurrence tables
- Occurrence from -750 to -450, Occurrence from -200 to -60

⇩ calc_igi_pri.py
- See Supplemental Figure S1

PRI table
- PRI200_60-750_450

Evaluation of sequences with a PRI table

Sequences to be evaluated + PRI table

⇩ chrom_scan.py

Scanned raw data ⇨ **evaluation**

⇩ peak_find_SG.py
- Smoothing with a bin 151 bp long & peak picking

Identification of PRI peaks in the evaluated sequences

⇩
- Threshold of peak height for TSS prediction are 0.0 for Arabidopsis and 0.06 for rice.
- Select the first peak upstream of CDS
- Expected TSS (Peak TSS of Top Genic TSS cluster) around the peak position (Arabidopsis: 0 bp ~ +200 bp; rice: -100 bp ~ +100 bp)

TSS prediction

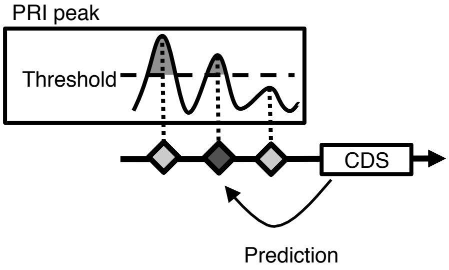

Supplemental Figure S12. Preparation and utilization of PRI
Sscripts and description of input and output data formats are avaiable at GifHub (https://github.com/yyyamamoto/TssPrediction).
